# The Link Between Sleeping and Type 2 Diabetes: A Systematic Review

**DOI:** 10.7759/cureus.48228

**Published:** 2023-11-03

**Authors:** Ali Darraj

**Affiliations:** 1 College of Medicine, Shaqra University, Shaqra, SAU

**Keywords:** hyperglycemia, sleep disorder, systematic review, type 2 diabetes, sleeping

## Abstract

Adults should get at least seven hours of sleep each night to preserve their overall health and well-being. Sleep disorders and other sleep-related issues affect a sizeable portion of the population. This reduction in sleep time may be brought on by the stress of modern life. This study's main goal was to look into the relationship between type 2 diabetes mellitus (T2DM) and sleep. In this study, papers were thoroughly screened utilizing keywords using databases like PubMed, PubMed Central, and MEDLINE. Additionally, a few articles were taken from the Cochrane Library. This study screened papers by title and abstract before applying inclusion/exclusion criteria. Eleven related studies were carefully assessed, and a quality evaluation check was conducted.

T2DM and sleep issues are frequent issues that frequently coexist. People with T2DM frequently experience sleep problems, which can be bad for their health, their mood, and their quality of life. On the other hand, sleep disturbances like obstructive sleep apnea increase the risk of metabolic diseases like T2DM. As part of standard clinical practice, all T2DM patients should be tested for sleep disturbances and given proper care. Evidence suggests that sleep problems may play a role in metabolic abnormalities as risk factors.

## Introduction and background

Sleep disorders and other sleep-related issues affect a sizeable portion of the population. This reduction in sleep time may be brought on by the stress of modern life. High blood sugar and insulin resistance are the two signs of type 2 diabetes mellitus (T2DM), a common chronic metabolic condition. The prevalence of type 2 diabetes has significantly increased globally during the past few decades. The primary objective of this study was to investigate the connection between type 2 diabetes and sleep. Four electronic databases were searched comprehensively for literature from their launch in 2013 without regard to language limitations. Of the 32 papers that the search turned up, 11 were included in the systematic literature.

The results of the study suggest that sleep, both in terms of quantity and quality, affects a patient's capacity to control their metabolism in type 2 diabetes. The studies showed that short sleepers had greater levels of circulating insulin during fasting, fasting glucose, and homeostatic model assessment for insulin resistance (HOMA-IR). Insufficient sleep and poor sleep hygiene were linked to increased glycated hemoglobin (HbA1c) levels in an adult type 2 diabetes study. In a research of middle-aged Caucasian volunteers, it was discovered that there was a substantial association between poor sleep quality and metabolic syndrome, as well as between sleep condition and insulin, fasting glucose levels, and insulin resistance. Type 2 diabetes and sleep disorders are prevalent conditions that often coexist. People with type 2 diabetes frequently experience sleep problems, which can have a detrimental effect on their general health, emotions, and quality of life.

High blood sugar and insulin resistance are the two signs of type 2 diabetes, a common chronic metabolic condition [1]. The prevalence of type 2 diabetes has significantly increased globally during the past few decades. A recent study has found a high correlation between sleep quality and the chance of acquiring type 2 diabetes [2], despite the fact that genetics and lifestyle factors like diet and exercise play a considerable role in the disease's development.

Sleep and phases

The two main phases of sleep, a complex physiological condition, are rapid eye movement (REM) sleep and non-REM (NREM) sleep [3,4]. There are three stages of NREM sleep, with stage three being the deepest and most rejuvenating. People repeatedly go through these stages during the night, with each cycle taking about 90 minutes [2].

Role of sleep in glucose regulation

Insulin Sensitivity

Sleep's impact on insulin sensitivity is one of the main factors tying it to type 2 diabetes. The pancreas secretes the hormone insulin, which promotes the uptake of glucose into cells and helps control blood sugar levels [5]. Reduced insulin sensitivity is a result of sleep deprivation, especially chronic sleep deprivation. As a result of the body's cells losing sensitivity to insulin, type 2 diabetes is thought to be characterized by an increase in blood sugar levels [6].

Circadian Rhythms

The circadian rhythm is a biological internal clock that controls a number of physiological activities, including glucose metabolism [3]. These circadian cycles can be disturbed by irregular sleep schedules or shift employment, which can then cause problems with glucose metabolism [7]. This disruption may lead to an elevated risk of type 2 diabetes and the emergence of insulin resistance.

Hormonal Regulation

Sleep is essential for controlling a number of hormones, including several that are directly connected to glucose metabolism [6]. For instance, lack of sleep can increase cortisol levels, which can induce insulin resistance and raise blood sugar levels. Additionally, growth hormones and other hormones that regulate glucose regulation are released during sleep [8].

The bidirectional relationship

There is a two-way association between sleep and type 2 diabetes. Diabetes itself can lead to sleep disruptions, just as poor sleep increases the likelihood of developing diabetes [9]. Diabetes patients frequently have symptoms including thirst and frequent urination, which can interfere with sleep [4]. Additionally, changes in blood sugar levels throughout the night may cause nocturnal awakenings and disturbed sleep [10]. The relationship between the duration of sleep and the prevalence of type 2 diabetes has been studied in many studies. The majority of adults require between seven and nine hours of sleep per night to remain healthy, despite the fact that everyone has a varied sleeping pattern. Type 2 diabetes has been linked to both short sleep length, which is often defined as less than six hours per night, as well as lengthy sleep duration, which is typically described as more than nine hours per night [11-13].

Literature review

The risk of type 2 diabetes is regularly increased by short sleep duration. This connection is influenced by a number of factors [12]. As an illustration, insufficient sleep can result in unhealthy eating habits and less physical exercise, both of which are risk factors for diabetes [14]. Additionally, a short sleep period throws off the body's hormonal balance, which raises the risk of insulin resistance [15]. Long periods of sleep have also been linked, though less frequently, to an increased risk of type 2 diabetes [16]. Long periods of sleep could be an indication of underlying health problems or poor sleep quality, both of which increase the chance of developing diabetes. Long periods of sleep can sometimes be a sign of abnormal sleeping patterns or underlying medical issues [9,17].

Not only does sleep duration matter, but also the quality of the sleep. When sleep is interrupted or of poor quality, even people who obtain enough sleep may be at risk. Sleep disturbances might worsen type 2 diabetes risk factors such as sleep apnea, restless leg syndrome, and insomnia [2,11,18]. Type 2 diabetes is more likely to develop in those who experience insomnia, which is characterized by difficulties falling or staying asleep [19]. Increased stress, hormone abnormalities, and poor glucose metabolism can all be caused by chronic sleeplessness. Type 2 diabetes and sleep have a complex and reciprocal relationship [15]. Sleep is essential for controlling how the body uses glucose, and irregular sleep patterns can raise the risk of developing diabetes and insulin resistance. On the other hand, diabetes itself has been linked to sleep issues, leading to a vicious cycle [9].

Prioritizing healthy sleep practices is crucial to reducing the risk of type 2 diabetes, including getting enough good sleep and keeping a regular sleep pattern [20]. Furthermore, those who have diabetes should be aware of the potential effects of their illness on sleep and seek appropriate therapy if they have sleep issues [21]. Knowing how closely type 2 diabetes and sleep are associated emphasizes the value of a holistic approach to health, which involves not just nutrition and exercise but also proper sleep hygiene and treatment of sleep-related problems [22]. Individuals can lower their risk of getting type 2 diabetes and enhance their general well-being by addressing all of these risk factors at once [23].

Cardiovascular dysfunction and metabolic mistakes can be the results of poor sleep [24]. All illnesses, especially chronic ones like diabetes, encourage emotional responses that can negatively impact sleep [25]. Frequent breathing pauses while you are asleep are a common sleep problem called sleep apnea [26]. It has been identified as a type 2 diabetes risk factor [27]. Insulin resistance and increased blood sugar levels can result from the occasional reductions in oxygen levels and sleep disruption brought on by sleep apnea [28]. The neurological condition known as restless leg syndrome makes people feel the need to move their legs constantly and is frequently accompanied by unpleasant feelings [29]. It may result in disturbed sleep, lowering the general level of rest, and maybe raising the risk of diabetes [3,6-8].

The effect of both the length and quality of sleep on health outcomes has been documented in multiple meta-analyses. The length of sleep and type 2 diabetes, obesity, hypertension, cardiovascular outcomes, and all-cause mortality were all examined in these research studies. Recent data from meta-analyses [22] evaluating the length and quality of sleep also point to the significant role that both sleep duration and quality play in metabolic function in patients with T2DM and as predictors of the onset of the disease. Extended or brief sleep duration, as well as erratic sleep-wake cycles, have been found to be strongly linked to elevated body mass index and compromised glycemic regulation [12].

In addition to its negative effects on sleep duration and quality, short or extended sleep duration also negatively affects quality of life in terms of health. In patients with T2DM, both excessive and insufficient sleep impairs glycemic control [20]. However, there is not enough evidence to back up a connection between T2DM and glycemic control, sleep length, or quality [26]. Thus, this study set out to perform a thorough assessment of the literature to provide more substantial evidence of the association between sleep and glycemic management in individuals with type 2 diabetes. This systematic review attempts to further examine any associations between type 2 diabetes and sleeping.

## Review

Methods

The systematic review for this investigation followed the Preferred Reporting Items for Systematic Reviews and Meta-Analyses (PRISMA) criteria [30].

Data Strategy

This study used the MEDLINE, Cochrane Library, PubMed, and PubMed Central databases. The terms "sleep deprivation" and "insulin resistance" were used in the search to find relevant papers. Then we used the Boolean operator "OR" to combine these concepts with keywords. As a result of the use of some keywords including "sleep," "diabetes," "type 2 diabetes," and "diabetes mellitus," PubMed search builders were developed, as shown in Table 1.

**Table 1:** Data sources and searches MeSH: Medical Subject Heading.

Topic	Keywords	Search
A link between sleeping and type 2 diabetes	Sleep, diabetes, type 2 diabetes	("sleep"[MeSH Terms] OR "sleep"[All Fields] OR "sleeping"[All Fields] OR "sleeps"[All Fields] OR "sleep s"[All Fields]) AND "type"[All Fields] AND "2"[All Fields] AND ("diabetes"[All Fields] OR "diabetes mellitus"[MeSH Terms] OR ("diabetes"[All Fields] AND "mellitus"[All Fields]) OR "diabetes mellitus"[All Fields] OR "diabetes"[All Fields] OR "diabetes insipidus"[MeSH Terms] OR ("diabetes"[All Fields] AND "insipidus"[All Fields]) OR "diabetes insipidus"[All Fields] OR "diabetic"[All Fields] OR "diabetics"[All Fields] OR "diabetes"[All Fields])

The keywords and their matched search builder were combined using the Boolean operator "OR" after being extracted from PubMed. Furthermore, restrictions on keywords-major themes were implemented. The final search strategy was created by combining all concepts and keywords with the Boolean operator "AND," as shown in Table 2.

**Table 2:** Search strategy in databases MeSH: Medical Subject Heading.

Search strategy	Articles
sleeping: "sleep"[MeSH Terms] OR "sleep"[All Fields] OR "sleeping"[All Fields] OR "sleeps"[All Fields] OR "sleep's"[All Fields] type 2 diabetes: "diabetes mellitus, type 2"[MeSH Terms] OR "type 2 diabetes mellitus"[All Fields] OR "type 2 diabetes"[All Fields]	Before filters applied = 105; after filters applied = 45 (filters: articles published in the last 10 years, articles published in the English language, free full text, adults)

Screening of Articles

We removed duplicates after acquiring the pertinent articles from all the databases. Following that, the papers were scrutinized based on their titles, abstracts, and readings of the whole text. Finally, 11 papers were chosen for further consideration and had their quality evaluated.

Inclusion and Exclusion Criteria

A search of the literature was done to find studies that looked at the relationship between type 2 diabetes and sleeping that were pertinent. Studies with an adult population and published as full-text publications in English during the previous 10 years were the inclusion criteria. Studies done on children and the elderly, as well as unpublished and gray literature, were disregarded.

Quality Appraisal Tools

Both factual analysis and the evaluation of the reliability of the evidence must be included in a systematic review. To assess the reliability of the data and the inherent biases of each study, the Critical Appraisal Skills Programme (CASP) technique must be used. By taking into account timeline creation, measurement errors, blinding, incomplete evaluations, selective efficacy of, and other biases, the proper risk of bias score was established. For systematic reviews and meta-analyses, the Cochrane risk of bias tool and the Assessment of Multiple Systematic Reviews 2 (AMSTAR 2) are used to assess the risk of bias and quality of included research, respectively, in randomized controlled trials (RCTs).

Results

**Figure 1:**
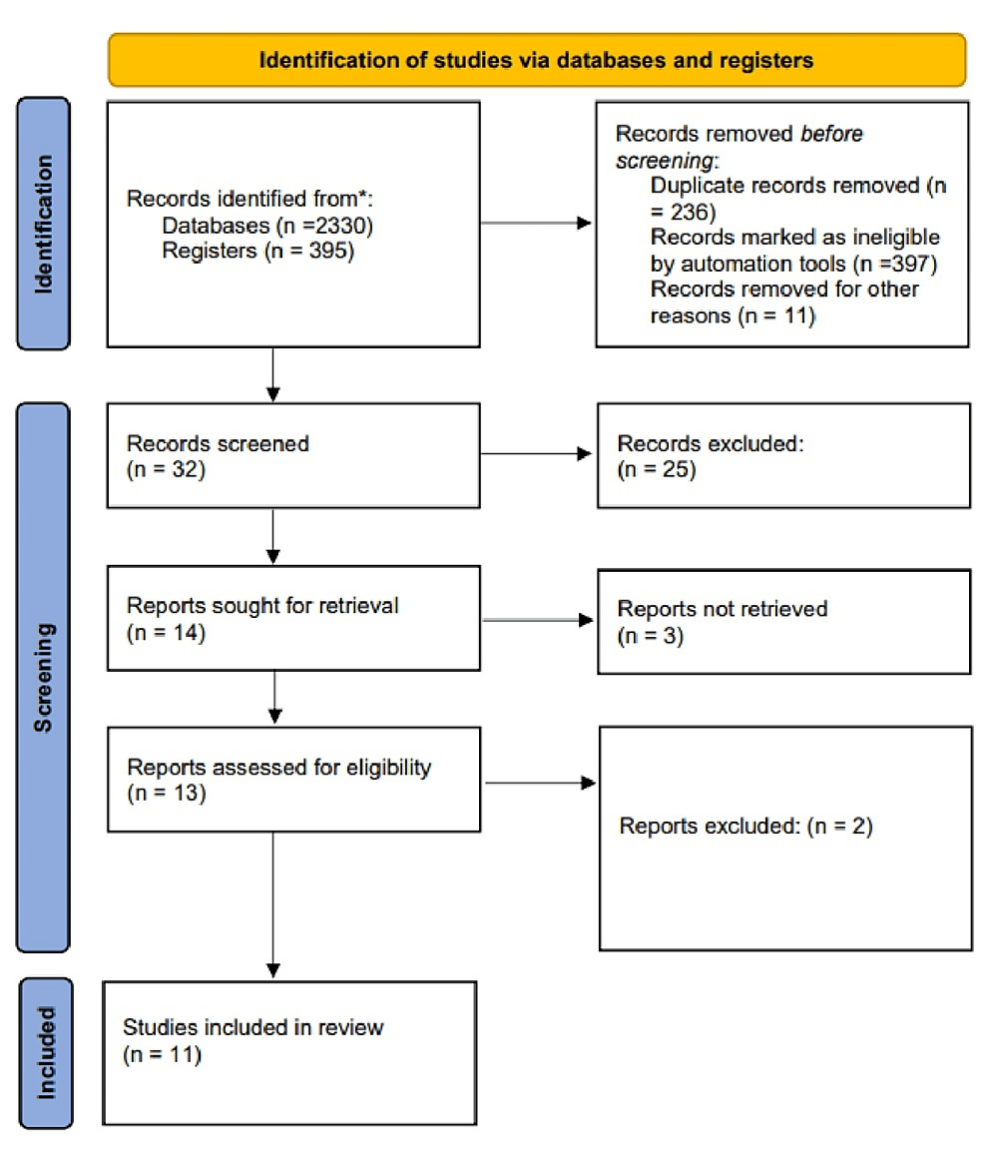
PRISMA flowchart of database searches and study selection PRISMA: Preferred Reporting Items for Systematic Reviews and Meta-Analyses.

A total of 395 different publications were created as a result of the study selection procedure. The methodical review, elimination, and article selection processes are depicted in Figure 1. A total of 32 articles were subjected to full-text analyses, while 14 papers were passed over after being evaluated solely by their titles or abstracts. Eleven studies were found when the exclusion criteria were applied, and their caliber was evaluated. Due to a lack of identifying information, one study was eliminated. It has already been highlighted in part how the complete synthesis of major issues perfectly satisfied the predefined outcomes of the current systematic review. Associations between type 2 diabetes and sleeping have all been chosen as crucial topics that pertain to the current systematic review's intended objectives (Table 3).

**Table 3:** Characteristics of included studies T2D: type 2 diabetes; OSA: obstructive sleep apnea; QOL: quality of life; PMB: portable multibiomedical; CPAP: continuous positive airway pressure; DM2: diabetes mellitus type 2; EB: emotional burden; SpO2: oxygen saturation.

No.	Author and year	Aim	Design	Population	Findings
1	Lee et al. (2023) [1]	How Asians' levels of sleep duration and the onset of diabetes were affected by their age, sex, and obesity.	Epidemiology study	Asians	The impact of sleep deprivation on the chance of developing T2DM during the length of the 16-year follow-up. Men and young, non-obese adults were the only ones who had this effect.
2	Bironneau et al. (2017) [2]	This cross-sectional study set out to find out whether patients with T2D have worse endothelial function as their OSA severity increases.	Cross-sectional	140 patients	Although moderate to severe obstructive sleep apnea (OSA) is relatively common, it has little effect on the function of the digital microvascular endothelial cells in T2D patients.
3	Nasir et al. (2022) [3]	To assess the link between quality of life and sleep in individuals with type 2 diabetes mellitus (T2DM), as well as its associated characteristics.	Cross-sectional	350 participants	According to this study, 32% of the population under study has poor sleep quality. Poor sleep quality is strongly correlated with nocturia, restless legs syndrome, and the EB component of type 2 diabetes (T2DM) discomfort.
4	Lou et al. (2012) [4]	The relationship between self-reported sleep length, sleep quality, and the prevalence of diabetes in an adult Chinese sample taken today.	Cross-sectional	Chinese adults	Short sleep length (6 hours) and poor sleep quality were both linked to a higher prevalence of diabetes, with higher rates among generally healthy Chinese individuals.
5	Hashimoto et al. (2020) [5]	The relationship between type 2 diabetes (T2D) patients' quality of life (QoL) and the sleep symptoms that lead to sleep disorders.	Cross-sectional	342 people with T2D	According to this study, sleep difficulties affect two-thirds of T2D patients. People who experienced frequent restroom breaks or excessive daytime sleepiness had a significantly lower quality of life than those who did not experience these symptoms.
6	Jain et al. (2017) [6]	Inpatients with type 2 diabetes will have their QOL and its factors evaluated as part of this cross-sectional comparative study.	Cross-sectional	50 patients	Patients with T2DM had lower QOL. The QOL of these patients is negatively impacted by a number of illness features. Regular sleeplessness may cause the QOL to further decline.
7	Lou et al. (2015) [7]	The purpose of this study is to examine sleep quality and quality of life in Chinese patients with type 2 diabetes mellitus (T2DM) and to evaluate the link between sleep quality and quality of life.	Cross-sectional	China adult	In T2DM, poor sleep is common and negatively correlated with quality of life. To improve diabetes patients' sleep quality, primary healthcare providers must incorporate sleep-related knowledge into diabetes self-management programs.
8	Li et al. (2016) [8]	The PMB recorder throughout a 24-hour period to examine how little sleep affects the blood pressure of people who put in a lot of extra work.	Cross-sectional	18 male	Lack of sleep may cause the sympathetic nervous system to become more active the next day, raising blood pressure. The PMB recorder proved effective for accurately assessing the association between ambient influences and blood pressure.
9	Ohkuma et al. (2013) [10]	The connection between type 2 diabetic patients' glucose levels, obesity, and sleep length.	Cross-sectional	4,870 Japanese	Independent of any relevant confounders, it has been demonstrated that sleep duration has a U-shaped relationship with obesity and HbA1c levels in type 2 diabetic patients. As a result, it may be a key modifiable component in the clinical care of these patients.
10	Martínez-Cerón et al. (2016) [9]	In patients with poorly controlled type 2 diabetes and OSA, the effect of CPAP on glycated hemoglobin (HbA1c) levels should be evaluated, and its causes should be determined.	Randomized clinical trial	50 patients	Compared to findings for a control group, continuous positive airway pressure (CPAP) therapy for six months improved glycemic control and insulin resistance in patients with poorly managed type 2 diabetes and OSA.
11	Gabryelska et al. (2021) [11]	The goal of the study was to evaluate how OSA patients' nocturnal oxygen saturation characteristics affected the emergence of DM2.	Cross-sectional	549 participants	While basal O2 is independent of apnea-hypopnea index (AHI), body mass index (BMI), and age as a predictor of DM2 in OSA patients, higher SpO2 nadir and basal SpO2 are connected with a delayed onset of DM2 in these patients.

Theme 1: Sleep Disturbances Associated With Diabetes

Multiple variables, such as nocturia, depression, restless legs syndrome, periodic actions of the limb, and nightly blood glucose changes that can result in hypoglycemia and hyperglycemia episodes, might contribute to insomnia in patients with diabetes. Depression is one of the major variables contributing to poor sleep in this population, and people with diabetes have a much higher chance of developing depression than people without diabetes [8]. Additionally, diabetes affects the central nervous system and changes neurobehavioral, neurotransmitter, and autonomic activities. It can also negatively affect endocrine systems, which results in sleep disorders [5].

For those who have diabetes, evaluation of sleep hygiene, sleep problems, and quality of sleep is crucial [2]. In fact, based on new research demonstrating a connection between sleep quality and glycemic management, sleep patterns and length are part of the comprehensive medical examination of diabetic patients [10].

Theme 2: Sleep Apnea and Diabetes

People with diabetes substantially more frequently experience sleep apneas, which are strongly correlated with obesity [7]. In actuality, there is a close connection between sleep-disordered breathing (SDB), decreased glucose tolerance, and obesity. Even while individuals with autonomic diabetic neuropathy have been observed to exhibit central-type apneas, including periodic breathing, obstructive sleep apnea (OSA) is still the most prevalent form of SDB [9]. Despite not being the primary cause of diabetes, sleep apnea increases insulin resistance even in those who do not already have the disease and who are not overweight [2,3]. According to Lou et al., type 2 diabetics may experience OSA in up to one in four cases, and a further quarter experience another sleep-related respiratory issue. Type 2 diabetes and OSA are more prevalent in overweight and obese people [7]. Weight apart from OSA still appears to have an effect on insulin resistance and glucose control. OSA causes sleep fragmentation, which interferes with slow-wave sleep and occasionally deprives the body of oxygen. Reduced glucose metabolism and insulin resistance are the results of these factors taken together [4,7,11].

Short-term treatment for sleep apnea appears to lower blood sugar and insulin resistance, according to Hashimoto et al. [5]. However, other investigations have not shown reductions in blood glucose levels after OSA therapy, leading some researchers to speculate that other factors, such as weight, may be the origin of the connection [1,3,5].

Theme 3: Sleep Disorder Management

Diabetes sleep disturbance is frequently complex in etiology. The history of sleep disorder should include factors such as trouble falling asleep or remaining asleep, excessive daytime sleepiness, snoring episodes as reported by spouses or family members, apneic episodes, and painful leg sensations that go away with movement in the evening [4,6,9].

Keeping a sleep diary for the previous two weeks might give you a thorough analysis of your sleep habits. All patients should undergo a brief clinical tool evaluation for depression [1,6,10]. With the right medical history and physical examination, the majority of sleep problems can be identified. However, to confirm the diagnosis of sleep apnea, a polysomnogram must be performed overnight. A primary insomnia diagnosis may be obtained if all medical and psychological causes of insomnia have been ruled out [5,9].

The treatment of sleeping problems is very rewarding. The treatment of common comorbid diseases as well as symptom reduction are frequently necessary for the management of sleep disorders [3]. The management of insomnia requires the application of behavioral measures, including adherence to excellent sleep hygiene, sleep restriction, cognitive behavioral therapies, and relaxation techniques [11]. For certain persons, pharmacological therapies, including the usage of benzodiazepine receptor agonists and more modern hypnotic medications like zolpidem and zaleplon, may be beneficial [4].

Discussion

Type 2 diabetes is frequently associated with short sleep duration. The results of the present study are consistent with the notion that both short and long sleep durations are associated with an elevated risk of T2DM. Different studies have discovered a link between the length of sleep and the onset of diabetes mellitus (DM). Female participants in a prospective analysis were found to have a higher risk of acquiring DM for both short and long sleep durations [22]. This connection persisted only for late sleepers after accounting for BMI. Another study discovered a statistically significant association between poor sleep quality and the emergence of incident T2DM in normal people [23].

In a second study, it was shown that males without diabetes who slept for less than six hours had a twice higher chance of developing diabetes, even after correcting for confounding variables [24]. Despite the fact that the majority of studies discovered a U-shaped relationship between sleep duration and the likelihood of getting diabetes, other studies indicated that only inadequate sleep was connected to a greater incidence of the disease [11,12]. A variety of glycometabolic markers, such as those for fasting glucose, post-load glucose, insulin sensitivity, and insulin secretion, were not examined for changes throughout the course of the 14-year study period. The pattern of changes in a number of glycemic markers may offer hints as to the mechanism behind the link between the quantity of sleep and the onset of diabetes [4,17].

Long-term sleepers also showed an increase in insulin resistance, although the pattern was also consistently observed in the other sleep duration groups. Scholars have postulated that the deterioration of pancreatic beta cell activity resulting from severe tiredness may be the cause of the increased risk of diabetes mellitus linked to extended sleep duration [26,27]. The main causes of type 2 diabetes are insulin resistance and a reduction in insulin. Previous cross-sectional research studies have connected diabetes-related markers to sleep duration. According to a study [22], getting less than seven hours of sleep per night was linked to a higher risk of diabetes. According to the study [19], short sleepers had greater levels of circulating insulin during fasting, fasting glucose, and HOMA-IR. Insufficient sleep and poor sleep hygiene were linked to increased HbA1c levels in an adult type 2 diabetes study [16]. In a research of middle-aged Caucasian volunteers, it was discovered that there was a substantial association between poor sleep quality and metabolic syndrome, as well as between sleep condition and insulin, fasting glucose levels, and insulin resistance [19].

In a different study, the etiology of the comorbid diseases that result from sleep loss is aided by the synthesis of serum amyloid A (SAA) during sleep restriction. In mice that were given sleep restrictions for 15 days or sleep deprivation for 72 hours, they discovered higher SAA levels. A noteworthy observation in those who experienced sleep deprivation was metabolic endotoxemia. Additionally, they discovered that following two nights of complete sleep deprivation, the plasma levels of SAA in healthy human subjects rose. The SAA levels stabilized after a single night of rest [20]. These results [7,23] imply that elevated SAA levels are probably a component of the pathophysiology connecting sleep loss to its numerous related diseases, including obesity and type 2 diabetes.

According to Bironneau et al., diabetes was linked to nearly regular reports of trouble falling asleep (21.1%), trouble staying asleep (21.9%), and excessive daytime sleepiness (12.2%). The existence of underlying SDB, nocturia, physical consequences of the disease, and underlying depression are frequently linked to sleep difficulties. In diabetic patients, polysomnography demonstrated increased alertness, a high number of awakenings, and fragmented sleep [3].

According to a study by Hashimoto et al., the circadian cycle lowers dynamic and static beta-cell function, which lowers glucose tolerance. Contrarily, circadian misalignment affected glucose tolerance, which was primarily brought about by reduced insulin sensitivity [5], with no impact on beta-cell activity. This might actually be a major contributing factor. The release of cortisol into the bloodstream is another frequent reaction to physiological stimuli [20].

It is also important to recognize a few of this study's flaws. First, certain research that was unpublished or published in other languages may not have been identified by this investigation. Furthermore, formal glucose tolerance testing was used to define diabetes in just one of the included studies. Second, because the majority of the included studies had insufficient data, we were unable to investigate how sleep duration and quality affected glycemic control in relation to population source, age group, and BMI. Third, the majority of studies only measured sleep duration once, thus it is possible that participants' sleep habits altered during the follow-up. As a result, it is possible that a single exposure measure will not capture all of the long-term impacts of sleep length on the chance of developing type 2 diabetes. Fourth, the absence of a meta-analysis of observational data precludes the direct accounting for residual or unmeasured confounding, even in the case of prospective data. Various confounders were attempted to be taken into account by adding adjusted estimates from multivariate models from each contributing paper. Although stratifying the studies by sex and race/ethnicity was not possible due to insufficient data from the original studies, this remains a relevant topic for future research. Finally, even though it was not looked at in this study, the quality of sleep, which is affected by things like sleep apnea, is a reliable indicator of the chance of developing diabetes.

With the exception of Lee et al.'s study, which discovered that extended sleep duration was not linked to an increased risk of diabetes, not many investigations looked into the connection between lengthy sleep duration and metabolic disturbances [1]. It is necessary to conduct more research to find out how lengthy sleep duration affects health. The majority of studies had a rather short follow-up period between the measurement of habitually short sleep and the occurrence of prediabetes. The lack of a suitable time interval for the onset of prediabetes may have an impact on the genuine nature of this association. It should be the goal of future research to lessen this selection bias and choose a group that is more representative of the world.

## Conclusions

In conclusion, type 2 diabetics frequently experience sleep disturbances, which have a detrimental impact on their health. To eventually improve health and, consequently, quality of life, efforts should be taken to diagnose and treat sleep disturbances in patients with type 2 diabetes, as doing so may prevent diabetes from progressing. In addition to directly impairing sleep due to nocturia, polyuria, diabetic neuropathy, and neuropathy pain, type 2 diabetes has also been linked to a number of chronic illnesses that can negatively impact sleep and quality of life, including OSA, cardiovascular complications, hypertension, cerebrovascular accidents, and depression. In a research of middle-aged Caucasian volunteers, it was discovered that there was a substantial association between poor sleep quality and metabolic syndrome, as well as between sleep condition and insulin, fasting glucose levels, and insulin resistance. Since insufficient and fragmented sleep can negatively impact a patient's quality of life, recovery, and ability to control their diabetes, healthcare providers treating patients with DM should pay special attention to sleep problems and the poor quality of life caused by these conditions. Another crucial tool in the diabetic control toolbox should be sleep education.
